# Trends over time in prescribing by English primary care nurses: a secondary analysis of a national prescription database

**DOI:** 10.1186/1472-6963-14-54

**Published:** 2014-02-06

**Authors:** Vari M Drennan, Robert L Grant, Ruth Harris

**Affiliations:** 1 Faculty of Health, Social Care & Education, Kingston University & St George’s, University of London, London, UK

**Keywords:** Non-medical prescribing, Nurse prescribing, Nurse roles, Primary care, Secondary analysis, Medication management

## Abstract

**Background:**

A growing number of countries legislate for nurses to have medication prescribing authority although it is a contested issue. The UK is one of these countries, giving authority to nurses with additional qualifications since 1992 and incrementally widened the scope of nurse prescribing, most recently in 2006. The policy intention for primary care was to improve efficiency in service delivery through flexibility between medical and nursing roles. The extent to which this has occurred is uncertain. This study investigated nurses prescribing activities, over time, in English primary care settings.

**Methods:**

A secondary data analysis of a national primary care prescription database 2006-2010 and National Health Service workforce database 2010 was undertaken.

**Results:**

The numbers of nurses issuing more than one prescription annually in primary care rose from 13,391 in 2006 to 15,841 in 2010. This represented forty three percent of those with prescribing qualifications and authorisation from their employers. The number of items prescribed by nurses rose from 1.1% to 1.5% of total items prescribed in primary care. The greatest volume of items prescribed by independent nurse prescribers was in the category of penicillins, followed by dressings. However, the category where independent nurse prescribers contributed the largest proportion of all primary care prescriptions was emergency contraception (9.1%). In contrast, community practitioner nurse prescribers’ greatest volume and contribution was in the category of gel and colloid dressings (27%), medicated stockings (14.5%) and incontinence appliances (4.2%). There were slightly higher rates of nurse prescribing in areas with higher levels of socio-economic deprivation and fewer physicians per capita, but the correlations were weak and warrant further investigation.

**Conclusions:**

The percentage of prescriptions written by nurses in primary care in England is very small in comparison to physicians. Our findings suggest that nurse prescribing is used where it is seen to have relative advantage by all stakeholders, in particular when it supports efficiency in nursing practice and also health promotion activities by nurses in general practice. It is in these areas that there appears to be flexibility in the prescribing role between nurses and general practitioners.

## Background

Access to safe and affordable medicines is one element of all countries’ strategies for ensuring public health [1]. Some countries have legislated for nurses to have authority to prescribe medicines, as part of national strategies for improving safety and efficiency in access to prescribed medicines, particularly in primary care settings and more countries are considering doing so [2]. The introduction of prescribing by nurses in most countries has been in response to either issues of shortages of physicians in rural and remote areas [3-8] or in response to perceived inefficiencies in primary health care delivery [9,10]. There is variation between countries as to the classes of medicines that nurses are authorised to prescribe and the qualifications of nurses with that authority. For example all registered nurses in South Africa can prescribe, but only for some classes of medicines, while in Australia only those with nurse practitioner qualifications can prescribe from a state- or territory-approved medicine list within their scope of practice [1].

The extent to which a health care innovation becomes integrated into usual practice depends on the interaction between features of the innovation, the adopter(s), and the context [11]. Government commissioned evaluations from Northern European countries at single points in time report the innovation of nurse prescribing as well received by patients, clinically appropriate and safe [12,13]. At a theoretical level the context for nurse prescribing is one of the shifting and contested boundaries of jurisdiction between professional groups in health care [14]. Prescribing by nurses’ remains a disputed innovation [15,16] including by some medical professionals [17-20]. At the same time, although nursing professional organisations in many countries have actively sought prescriptive authority [21-24], some nurses have expressed concerns regarding appropriate training, support and remuneration for this role [25-28] and ambivalence to a more medically orientated role [29-31]. The contextual influences are demonstrated by the variation in rates of self-reported levels of use of prescribing authority by nurses. Surveys of nurse practitioners in the United States of America report over 90% regularly using their authority to prescribe [32,33], in comparison to a survey of Australian nurse practitioners with endorsement to prescribe, which found that 41% did not prescribe [34]. The adoption of health care innovation also changes over time. Studies of nurse prescribing to date have drawn prescribing evidence at single points in time or from aggregated data [12,13,35,36], often in relation to programmes targeted at specific patient populations [5,37] or particular types of medicines such as analgesics or antidepressants [38,39].

This study investigated the extent of the use of prescribing authority by primary care nurses over five years in the context of English government health policies which supported greater flexibility in health professional roles to improve access and efficiency in primary care [40,41]. An incremental history of legislation and implementation in England [42-47] culminated in 2006 with registered nurses with additional independent prescribing qualifications being authorised to prescribe any licensed medicine, including some Controlled Drugs, for any medical condition within their clinical competence and scope of practice (see Table 1).

**Table 1:** Legislative authority and NHS mechanisms for nurses to prescribe medicines in the UK from 1992

**Year**	**Legislation and NHS mechanisms**
**1992**	Legislation for specialist qualified community nurses (district nurses and health visitors) with extra prescribing qualifications to prescribe from a limited nurse formulary [42].
**1996**	Legislation for a limited Nurse Prescribers’ Formulary for district nurses and health visitors, which included dressings, medicines for skin conditions and catheter management [43].
**1998**	The National Health Service (NHS) Executive authorised a national introduction of nurse prescribing by district nurses and health visitors, with additional prescribing qualifications, using the Nurse Prescribers’ Formulary for District Nurses and Health [44].
**2001**	Legislation passed for the extension of prescribing authority to nurses, midwives and health visitors, with additional qualifications as independent prescribers and supplementary prescribers (i.e. nurses with additional qualifications given authority to prescribe from a patient specific medicines list prescribed by a medical or other independent prescriber) [45].
**2002**	The NHS introduced. The Nurse Prescribers extended formulary (NPEF) list, including 140 prescription only medicines (POMs) all general sales list pharmacy medicines, for independent nurse prescribers undertaking an extended prescriber qualification [46,47].
**2006**	Legislation for nurse independent prescribers to prescribe any licensed medicine including some Controlled Drugs, for any medical condition within their clinical competence [48].

This incremental history has also resulted in two types of qualification for prescribing by nurses [48]:

• An independent prescribing qualification for nurses (INP) to prescribe, within their scope of practice, any licensed medication including some controlled drugs [48]. Registered nurses, with more than three years clinical practice, can with their employers support undertake a Nursing and Midwifery Council (NMC) approved, theoretical (minimum of 26 days) and supervised practise (12 days) course for independent prescribing. The practice element includes supervision and assessment by a designated medical practitioner [49]. The course is at degree level or may be part of masters programmes.

• A community practitioner nurse prescriber (CPNP) qualification to prescribe from a limited formulary [48]. Registered nurses working or intending to work in primary care or community can undertake a NMC approved course as part of a degree level specialist community qualification or as a standalone course [49]. This nurse formulary is a nationally agreed limited list including items such as emollients, laxatives, anti-fungal preparations, some analgesics (e.g. paracetamol, aspirin, ibuprofen), nicotine replacement products, parasiticidal preparations, and wound management products, catheters and catheter management preparations [50].

A previous systematic review [51] and subsequent updating found no United Kingdom (UK) studies that reported from objective prescribing data by nurses in primary care (although as the review noted some studies may have included data from primary care but it was not possible to separate from that in hospital settings). This study addressed the following research questions:

• What percentage of nurses, with authorisation to prescribe in primary care, used their prescribing authority and has this changed over the first five years of independent nurse prescribing?

• What types of medications are prescribed in the greatest volume by nurse prescribers with authorisation to prescribe in primary care?

• For what types of medications do nurse prescribers with authorisation contribute the greatest proportion of primary care prescriptions, and does this suggest any flexibility between the roles of physicians and nurses?

• Are there primary care contextual circumstances in which nurses are more actively prescribing medicines?

## Methods

The study design was a secondary data analysis of the administrative records of National Health Service (NHS) primary care prescriptions and contextual primary care environment and workforce data in England available in the public domain.

### Setting

The NHS is a tax funded health service which provides for universal registration as a patient with primary care general practitioners (GPs) [52]. GPs are known as family physicians in some countries. GPs employ practice nurses to work in their practices or surgeries. Their patients are also provided services by district nurses (known as home visiting nurses in some countries) and health visitors (known as public health nurses in some countries). These nurses were employed by community health services in local area NHS organisations, which were called Primary Care Trusts (PCTs) at the time of the study. In 2010, there were 154 PCTs in England with a median patient population of 298,391 (quartiles 227,944 and 431,018) registered with GPs [53]. Within England there are two prescribing qualifications [47,48] of interest in this study:

• An independent prescribing qualification for nurses (INP) to prescribe, within their scope of practice, any licensed medication including some controlled drugs.

• A community practitioner nurse prescriber (CPNP) qualification to prescribe from a limited formulary.

### Sample

Every NHS prescription issued and dispensed for a general practice patient is entered onto the administrative database of the NHS Business Service Authority called ePACT (electronic Prescribing Analysis and Cost). The database both identifies the prescriber by a unique identifying code and also links the prescription to the GP with whom the patient is registered. Nurse prescribers have to be authorised by their employer to be registered and receive a unique identifying number with ePACT. The ePACT database is comprehensive in managing NHS primary care prescribing and dispensing costs across England.

### Data collection

Data on all prescriptions (irrespective of type of prescriber) were obtained from ePACT under the terms of the Freedom of Information Act 2000 [54] from October 2006 to September 2010. The ePACT data obtained provided the following variables: the number of items prescribed by the categories in the British National Formulary (BNF) [53], the location by the PCT, the type of prescriber (GP or nurse), and for nurses their qualification (independent or community prescriber) and employer (PCT or a GP). Data on the primary care context were obtained from the NHS Information Centre [50] for each PCT area. This included the following data: percentage of GPs per 1000 capita, percentage of single handed (i.e. a solo medical practitioner) general practices, percentage of general practice patients aged under 15, and percentage of general practice patients aged over 65. In addition a measure of socio-economic deprivation, the Index of Multiple Deprivation [55], was obtained for each PCT [56].

We also obtained the number of nurses GPs and nurses registered with ePACT at 1 April each year from 2006 to 2010 inclusive.

### Ethical considerations

Ethical approval was not required for this secondary analysis of organisation-level data in the public domain.

### Data analysis

Data were analysed descriptively using Stata software, version 11 [57]. When calculating nurse prescriptions as a proportion of all primary care, categories of drugs and devices were included only if more than 10,000 items were prescribed in total over the five years. As the data represents every primary care prescription in England during the time period, the only role of inferential statistics would be in extrapolating to the future or comparing specific organisations, which was not our aim.

There were a small number of ePACT records where the profession or the location was unknown, which were only included in overall national statistics. For this analysis, we omitted those prescribers with only one prescription in a year to avoid coding errors. A limitation of ePACT data in the public domain is that individual patients and prescribers are not identified. Consequently prescriptions made for, or by, the same person cannot be linked.

## Results

**Figure 1:**
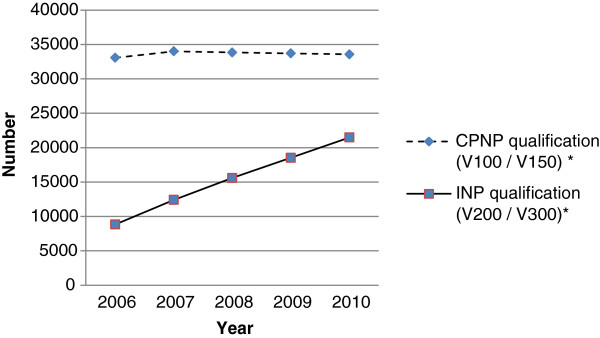
Trends in numbers of nurses with prescribing qualifications (UK) 2006-2010*. *Data source: Nursing and Midwifery Council [40] and personal communication from N. Rossi, NMC communications officer to R.L. Grant. 2011.

Between 2006-2010 the number of nurses registered by their employers to prescribe with ePACT rose by 18% from 30,753 to 36,281 (Figure 1). The greatest increase was those with independent nurse prescribing qualifications (INPs) from 5014 to 12975 while the Community Practitioner Nurse Prescribers (CPNPs), who use a limited formulary, decreased by 2437.

**Figure 2:**
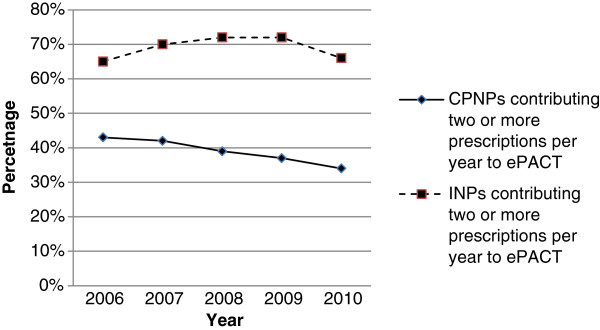
Trends in percentage of primary care nurses, registered on ePACT, prescribing 2006-2010.

While the numbers of nurses actively prescribing, as recorded on ePACT by 2 or more prescriptions in a year, rose in the time period by 18% from 13,391 to 15,841, this remained at 43% of all those nurses registered with ePACT to prescribe. A greater percentage of those with independent nurse prescribing qualifications registered with ePACT were actively prescribing in the time period (from 65% to 72% annually) than CPNPs, who decreased as active prescribers between 2006 and 2010 (Figure 2).

Nurses prescribed 1.2% of all items on ePACT during the time period rising from 1.1% (8,760,634/773,090,199) in 2006-7 to 1.5% (13,573,943/907,152,654) in 2009-10.

### Trends in the types of medicines and medicinal products prescribed by nurses

The volume of prescriptions written by nurses varied between the different BNF [50] categories of medicines and medicinal products. In 109 of 771 BNF [50] categories there were no nurse prescriptions at all. The greatest volume of items prescribed by nurses is given in Table 2 (with data for more categories provided in Additional file 1: Table S1) demonstrating both the different types of medicine categories those with different qualifications can prescribe from and percentage of the total prescribed in the period in general practice.

**Table 2:** The five BNF categories from which nurses prescribed the most items 2006-2010 (descending order)

	**INPs**			**CPNPs**		
	***Category***	***Number of items***	**% *of all items prescribed in primary care***	***Category***	***Number of items***	**% *of all items prescribed in primary care***
1	*Penicillins*	3,773,977	4.05%	*Dressings*	7,700,458	16.32%
2	*Dressings*	2,303,643	4.88%	*Devices*	1,314,417	2.68%
3	*Adrenoceptor Agonists*	1,642,286	1.61%	*Emollients*	909,523	1.43%
3	*Non*-*Opioid Analgesics*	1,593,641	0.88%	*Incontinence appliances*	313,654	4.24%
5	*Devices*	1,410,479	2.88%	*Stoma appliances*	305,767	2.28%

The BNF [50] categories where nurses prescribed more than 10% of the total items prescribed in primary care over 5 years were all among wound dressings, devices and incontinence or stoma appliances (Table 3 with further data presented in Additional file 2: Table S2). In the 32 BNF [50] categories of wound dressings, incontinence and stoma appliances associated devices nurses prescribed more than 20% of the total items prescribed in these categories over the time period.

**Table 3:** The five BNF categories where nurses make the greatest contribution to prescribing in primary care 2006-2010 (descending order)

	**INPs**			**CPNPs**		
	***Category***	***Number of items***	**% *of all items prescribed in primary care***	***Category***	***Number of items***	**% *of all items prescribed in primary care***
1	*Emergency Contraception*	123,082	9.06%	*Gel And Colloid Dressings*	22891	26.98%
2	*Drugs for threadworms*	50,673	5.15%	*Dressings*	7,700,458	16.32%
3	*Medicated stockings*	6,807	5.12%	*Alcohols and saline*	56,191	16.02%
4	*Oils*	43,950	5.00%	*Medicated stockings*	19,314	14.52%
5	*Dressings*	2,303,643	4.88%	*Incontinence appliances*	313,654	4.24%

The BNF [50] categories which had the greatest increase in nurse prescribing over the study period (difference in percentage of items between the first and last 6 months of the 5-year period) included, apart from wound dressings and incontinence and stoma devices, were preparations for de-sloughing wounds (16%), emergency contraception (8%), penicillins (6%), preparations for vaginal and vulval infections (5%), and preparations for cuts and abrasions (5%) e.g. cetrimide cream, flexible collodion.

### Primary care context and prescribing

In 2010 general practice employed independent nurse prescribers (INPs) contributed up to 2% of all items prescribed. In two PCT areas general practice-employed INPs prescribed more than 3% of items, and one PCT area had over 5%. However, there were no prescriptions recorded from INPs employed in general practices in 8% (13 of 154) of PCTs. Independent nurse prescribers employed by PCTs contributed under 0.5% of items prescribed in most PCTs. Nineteen PCTs had employed INPs who contributed higher percentages of items. Only in 3 PCTs was this over 1% (1.3%, 1.3% and 2.1%).

**Figure 3:**
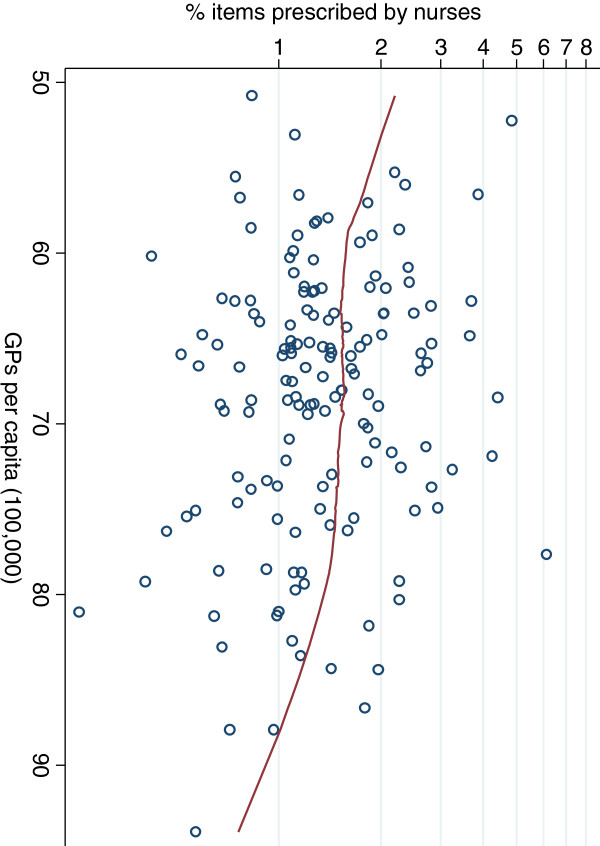
Percentage of items prescribed by nurses in relation to the number of GPs per population capita.

**Figure 4:**
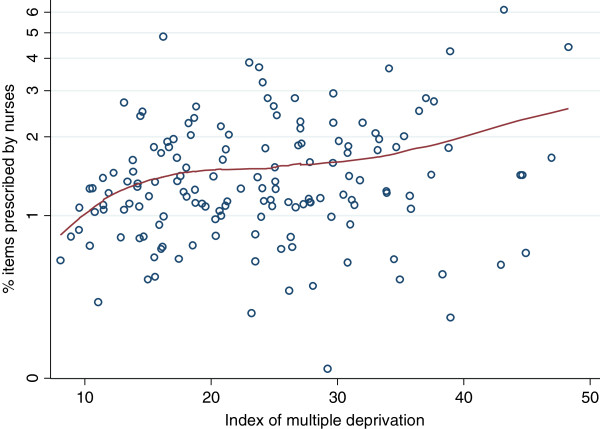
Percentage of items prescribed by nurses in relation to the PCT Index of Multiple Deprivation.

The level of nurse prescribing in different PCT areas was not obviously related to the general practitioner variables or patient age distribution, except for an association between the number of GPs per capita and nurse prescribing. Figure 3 presents a smoothed (lowess) regression line relating the GPs per capita to the percentage of items prescribed by nurses, on a logarithmic scale. This suggests that the PCT areas with the fewest GPs per capita have nurses who prescribe approximately double the percentage of items than the PCT areas with the most GPs (Spearman’s rho = -0.16). However, the trend was only apparent at the extremes and there was a great deal of unexplained variation between PCT areas, so it cannot be regarded as more than tentative evidence for increased nurse prescribing where there are fewer general practitioners per capita. Although there was a weak positive correlation between higher PCT deprivation and more GPs per capita (Spearman’s rho = 0.22), we also found that in more deprived PCT areas there were higher proportions of items prescribed by nurses (Figure 4; Spearman’s rho = 0.19).

## Discussion

This study has used NHS prescription administrative databases to examine trends in prescribing practices of 30,753 (2006) rising to 36,281 (2010) primary care nurses in England. To our knowledge this study presents data on the prescribing practices of the largest number of primary care nurses to date.

We found that NHS employers authorised greater numbers of primary care nurses to prescribe over the time period. The percentage actually prescribing as recorded by the prescription administrative system remained static over time (43%). We have objectively quantified the percentages of nurses that are using their independent nurse prescriber (INP) qualification and demonstrated a decline in use of community nurse prescribing (CNP) qualifications, not reported previously. We found higher percentages of nurses in primary care not using their prescribing qualifications than reported by generic surveys, including primary care, in the USA [32,33] and the UK [12,29] but lower than Australia [34] where prescribing rights have been more recently introduced. The finding of regional variation, with areas with no prescribing by practice employed nurses, has been noted before [58] but the evidence here suggests this feature has not changed over time.

These findings suggest that this innovation has yet to be fully adopted as usual or normal practice for primary care nurses with prescribing qualifications [11]. Some UK studies have suggested nurses’ lack of employer support [30,59] and there is a need for change champions in local health care systems [60], however these were nurses who had employer support as evidenced by the employer registering them as prescribers with the ePACT database. It raises questions as to why such numbers of nurses have gained the prescribing qualification for it then not to be used in practice. One hypothesis, derived from the literature [30,59] and requiring testing, is that nurses who have access to a clinical mentor or supervisor are more likely to prescribe than those who no or little access. A second hypothesis that requires further investigation is that the ambivalence to this role is more wide spread amongst primary care nurses than previously reported and they are choosing not to prescribe and as such acting as ‘*street*-*level bureaucrats*’ [61] i.e. as front line staff making policy through their implementation decisions. Primary care nurses acting as street-level bureaucrats in the face of policy implementation has been noted before in the UK [62] and other countries [63].

While the number of nurses and the volume of prescribing by nurses increased over the five year period, prescribing in primary care remains an activity mainly undertaken by doctors in England. This has been noted previously [64] and in other countries where nurses prescribe in primary care [36]. We found that the largest volume of items prescribed by nurses in primary care (both INPs and CNPs) were those items used in common nursing care activities practice i.e. wound dressings, incontinence and stoma devices. Beyond these, the medicine categories where there had been the greatest increase in both volume and percentage of prescribing compared to GPs, were those that could be bracketed as health promotion e.g. contraception, smoking cessation [65]. While practice nurses have become involved in the chronic disease management processes for primary care patients [66,67] it is not evident from this study that as a group they undertake significant prescribing with these patients. Surveys of practice nurses in the UK over the last two decades show that health promotion and family planning activities are ranked as the most frequent [68,69]. One hypothesis, that requires further investigation, is that nurse prescribing is most acceptable to both nurses and others when it improves their efficiency in delivering primarily nursing interventions, treatments or health promotion within their scope of practice. Such investigation could include the prescribing practices of nurses with specific responsibilities for patients with long term conditions such as community matrons in the English setting [70].

We found tentative evidence that there may be higher rates of nurse prescribing in areas with lower ratios of GPs to patient populations (Spearman’s rho = -0.16) and higher levels of deprivation (Spearman’s rho = 0.19). We suggest this requires further investigation over time and in the face of predicted shortages of GPs in deprived and rural areas, where the UK and other countries have difficulty in attracting and retaining family doctors [71,72].

Our findings of the first five years following the introduction of independent nurse prescribing, involving all classes of medicines, suggests that while the English policy objectives were for increased flexibility professional roles [40,41], this has only been at the margins of medical practice. However, it may have significantly improved access and efficiency in health care for some groups of patients and released medical time. Prescribing by nurses in primary care for specific patient groups has the potential to release general practitioner time. Economic modelling, from one UK study, for patients with infections and those with hypertension, suggested the involvement of independent nurse prescribers was less expensive compared to a GP only prescribing model [12]. Further investigation is required over longer periods and specifically examining questions of efficiency, improved access and cost effectiveness for different patient populations.

The study has a number of limitations. As an analysis of a data set established for financial reimbursement, it cannot investigate at the patient or prescriber level. Hence, we were not able to address other aspects of nurse prescribing activities such as ceasing medications. Nor is it able to address questions such as clinical safety or health economics. The data are limited to five years of prescriptions in NHS England and other mechanisms which circumvent the ePACT database may be in place such as patient group directions [73] (known elsewhere by terms such as ‘standing orders’ [74]) with bulk purchase e.g. vaccines, masking a greater level of nurse activity in prescribing. However, despite these limitations this study provides empirical data and insights not available elsewhere as to the types of prescribing undertaken by primary care nurses, over time and from a national perspective. As such it offers some valuable information to nurses and policy makers both in the UK and elsewhere and in addition sets a research agenda for future study.

## Conclusions

The percentage of prescriptions written by nurses in primary care in England is very small in comparison to general practitioners and there has been little change in that over five years. The adoption of any innovation in health care systems is influenced by a range of contextual and individual factors. Our findings suggest that nurse prescribing has been most frequently used in situations where it is seen to have relative advantage by all stakeholders, in particular when it supports efficiency in nursing practice, most commonly of wound and incontinence management but also the health promotion activities of nurses in general practice. It is in these areas that there appears to be flexibility in the prescribing role between nurses and general practitioners.

## Competing interests

The authors declare that they have no competing interests.

## Authors’ contributions

VMD conceived, designed, undertook interpretation and wrote the first draft of the paper. RG designed, undertook statistical analysis, interpretation and made intellectual contribution in the drafting of the paper. RH designed, undertook interpretation and made intellectual contribution in the drafting of the paper. All authors read and approved the final manuscript.

## Pre-publication history

The pre-publication history for this paper can be accessed here:

http://www.biomedcentral.com/1472-6963/14/54/prepub

## Supplementary Material

Additional file 1: Table S1 The 20 British National Formulary categories from which nurses prescribed the most items, 2006-2010 (descending order), by prescribing qualification. Click here for file

Additional file 2: Table S2 The 20 British National Formulary categories where nurses made the greatest contribution to prescribing in primary care, 2006-2010 (descending order), by prescribing qualification. Click here for file
