# Supplementary material for: Trends over time in prescribing by English primary care nurses: a secondary analysis of a national prescription database
Source: BMC Health Serv Res. 2014 Feb 6;14:54. doi: 10.1186/1472-6963-14-54 (PMC3922985; doi:10.1186/1472-6963-14-54)
Supplement: Additional file 1: Table S1 — The 20 British National Formulary categories from which nurses prescribed the most items, 2006-2010 (descending order), by prescribing qualification. [file 1472-6963-14-54-S1.docx]

Additional file 1

The 20 British National Formulary categories from which nurses prescribed the most items, 2006-2010 (descending order), by prescribing qualification

|  | **Independent Nurse Prescribers** | | | **Community Practitioner Nurse Prescribers** | | |
| --- | --- | --- | --- | --- | --- | --- |
|  | **Category** | **Number of items** | **% of all items prescribed in primary care** | **Category** | **Number of items** | **% of all items prescribed in primary care** |
| 1 | Penicillins | 3,773,977 | 4.05% | Dressings | 7,700,458 | 16.3% |
| 2 | Dressings | 2,303,643 | 4.88% | Devices | 1,314,417 | 2.68% |
| 3 | Adrenoceptor agonists | 1,642,286 | 1.61% | Emollients | 909,523 | 1.43% |
| 4 | Non-opioid analgesics | 1,593,641 | 0.88% | Incontinence appliances | 313,654 | 4.24% |
| 5 | Devices | 1,410,479 | 2.88% | Stoma appliances | 305,767 | 2.28% |
| 6 | Combined hormonal contraceptives/systems | 1,333,860 | 4.68% | Drugs used in substance dependence | 261,234 | 1.06% |
| 7 | Corticosteroids (respiratory) | 1,290,583 | 1.65% | Antifungal preparations | 143,936 | 1.45% |
| 8 | Topical corticosteroids | 1,271,840 | 2.05% | Barrier preparations | 118,702 | 2.81% |
| 9 | Emollients | 1,257,299 | 1.98% | Local anaesthetics | 109,097 | 2.30% |
| 10 | Drugs used in substance dependence | 1,148,483 | 4.65% | Non-opioid analgesics | 107,682 | 0.06% |
| 11 | Renin-angiotensin system drugs | 1,095,126 | 0.45% | Osmotic laxatives | 67,957 | 0.19% |
| 12 | Lipid-regulating drugs | 1,071,490 | 0.43% | Antifungal drugs | 65,903 | 0.76% |
| 13 | Non-steroidal anti-inflammatory drugs | 935,650 | 1.10% | Alcohols and saline | 56,191 | 16.02% |
| 14 | Antidiabetic drugs | 851,075 | 0.83% | Oropharyngeal anti-infective drugs | 50,114 | 3.67% |
| 15 | Antihistamines | 779,037 | 1.59% | Parasiticidal preparations | 32,814 | 1.82% |
| 16 | Sulphonamides and trimethoprim | 717,819 | 4.44% | Diabetic diagnostic and monitoring agents | 29,426 | 0.10% |
| 17 | Proton pump inhibitors | 635,626 | 0.40% | Gel and colloid dressings | 22,891 | 27.00% |
| 18 | Macrolides | 613,191 | 2.98% | Medicated stockings | 19,314 | 14.52% |
| 19 | Antibacterials | 609,931 | 4.45% | Stimulant laxatives | 15,799 | 0.06% |
| 20 | Antiplatelet drugs | 577,674 | 0.32% | Parenteral preparations for fluid and electrolyte imbalances | 8,491 | 1.08% |
