# Supplementary material for: Trends over time in prescribing by English primary care nurses: a secondary analysis of a national prescription database
Source: BMC Health Serv Res. 2014 Feb 6;14:54. doi: 10.1186/1472-6963-14-54 (PMC3922985; doi:10.1186/1472-6963-14-54)
Supplement: Additional file 2: Table S2 — The 20 British National Formulary categories where nurses made the greatest contribution to prescribing in primary care, 2006-2010 (descending order), by prescribing qualification. [file 1472-6963-14-54-S2.docx]

Additional file 2

The 20 British National Formulary categories where nurses made the greatest contribution to prescribing in primary care, 2006-2010 (descending order), by prescribing qualification.

|  | **Independent Nurse Prescribers** | | | **Community Practitioner Nurse Prescribers** | | |
| --- | --- | --- | --- | --- | --- | --- |
|  | **Category** | **Number of items** | **% of all items prescribed in primary care** | **Category** | **Number of items** | **% of all items prescribed in primary care** |
| 1 | Emergency contraception | 123,082 | 9.06% | Gel and colloid dressings | 22,891 | 26.98% |
| 2 | Drugs for threadworms | 50,673 | 5.15% | Dressings | 7,700,458 | 16.3% |
| 3 | Medicated stockings | 6,807 | 5.12% | Alcohols and saline | 56,191 | 16.02% |
| 4 | Oils | 43,950 | 5.00% | Medicated stockings | 19,314 | 14.52% |
| 5 | Dressings | 2,303,643 | 4.88% | Incontinence appliances | 313,654 | 4.24% |
| 6 | Vaginal and vulval infections | 238,723 | 4.78% | Oropharyngeal anti-infective drugs | 50,114 | 3.67% |
| 7 | Combined hormonal contraceptives / systems | 1,333,860 | 4.68% | Barrier preparations | 118,702 | 2.81% |
| 8 | Minor cuts and abrasions | 11,141 | 4.66% | Devices | 1,314,417 | 2.68% |
| 9 | Drugs used in substance dependence | 1,148,483 | 4.65% | Local anaesthetics | 109,097 | 2.30% |
| 10 | Antibacterials | 609,931 | 4.45% | Stoma appliances | 305,767 | 2.28% |
| 11 | Sulphonamides and trimethoprim | 717,819 | 4.44% | Parasiticidal preparations | 32,814 | 1.82% |
| 12 | Otitis externa | 427,205 | 4.42% | Bladder instillations | 597 | 1.71% |
| 13 | Penicillins | 3,773,977 | 4.05% | Antifungal preparations | 143,936 | 1.45% |
| 14 | Antibacterial preparations | 304,285 | 3.93% | Emollients | 909,523 | 1.43% |
| 15 | Progestogen-only contraceptives | 539,334 | 3.89% | Parenteral preparations for fluid and electrolyte imbalances | 8,491 | 1.08% |
| 16 | Gel and colloid dressings | 3,062 | 3.61% | Drugs used in substance dependence | 261,234 | 1.06% |
| 17 | Urinary tract infections | 130,844 | 3.59% | Oils | 7,844 | 0.89% |
| 18 | Parasiticidal preparations | 63,816 | 3.53% | Antifungal drugs | 65,903 | 0.76% |
| 19 | Spermicidal contraceptives | 2,019 | 3.38% | Preparations for eczema | 1,324 | 0.48% |
| 20 | Aromatic inhalations | 3,277 | 3.26% | Drugs for threadworms | 4,576 | 0.47% |
